# Prevalence of prehospital pain and pain assessment difference between patients and paramedics: a prospective cross-sectional observational study

**DOI:** 10.1038/s41598-024-56072-8

**Published:** 2024-03-07

**Authors:** David Häske, Wolfgang Dorau, Fabian Eppler, Niklas Heinemann, Florian Metzger, Benjamin Schempf

**Affiliations:** 1 Center for Public Health and Health Services Research, University Hospital Tübingen, Osianderstrasse 5, 72076 Tübingen, Germany; 2 German Red Cross, Emergency Medical Service, 72764 Reutlingen, Germany

**Keywords:** Health services, Public health

## Abstract

Adequate analgesia is one of the most important interventions in emergency medicine. However, studies suggest that many patients are undertreated for pain. There can be many reasons why patients assess their pain differently to the paramedics. This study aimed to assess the differences in pain ratings between patients and paramedics and factors influencing them in prehospital emergencies. This prospective cross-sectional observational study included patients treated and transported by paramedics or paramedics and emergency physicians of the German Red Cross in Reutlingen, Germany. This study included 6,365 patients. The prevalence of pain was 49.7%. Among patients with a self-reported numerical rating scale (NRS) > 0, the mean patient pain rating was NRS 4.2 ± 2.7, while the mean paramedic pain rating was NRS 3.6 ± 2.4 (*p* < 0.001). Approximately 11.8% (n = 751) of patients reported subjectively unbearable pain. Patients reported a mean NRS of 7.7 ± 1.8 for unbearable pain, but a mean NRS of 3.3 ± 2.0 for bearable pain (*p* < 0.001). The difference in pain ratings between patients and paramedics increased with pain severity. Univariate analysis showed that there were no gender differences, but the difference in pain rating was influenced by patient age (*p* < 0.001) and paramedic age (*p* = 0.042). The differences in pain ratings were particularly pronounced for abdominal diseases (*p* < 0.001) and trauma (*p* < 0.001). There is a difference in pain ratings between patients and paramedics, which increases with pain severity and appears to be associated with the patient’s age and the paramedic’s age. To determine the desire and need for analgesics, the question about unbearable pain is a good addition to the NRS.

## Introduction

Adequate analgesia is recognized as one of the most crucial and frequently employed interventions in emergency medical services (EMS) and emergency departments^1–3^. Despite pain management being a fundamental human right, studies indicate that many patients receive inadequate pain treatment^4–6^. One contributing factor is the subjective nature of pain, which results in differential perceptions and experiences between healthcare professionals and patients^7^. Older patients with greater pain experience often exhibit different pain assessments than “inexperienced” patients^8^. Older patients, because of their greater life experience, have generally experienced more pain, whether it is chronic pain, acute pain from injury and illness, or medical interventions^9^.

However, pain is not only influenced by life experience but also by gender, ethnic factors, etc.^9–11^. Lvovschi et al. note that the cognitive functions of the paramedics, as well as culture, age, own pain experience, etc., depending on their work context (pain education, overcrowding, medical-economic context, etc.) are also relevant to the interaction between healthcare professionals and patients ^12^. On the other hand, there is the patient’s cultural background, cognitive abilities, and level of education, which must be contextualized for the modalities of verbal expression of pain. Proposals for a “Multimodal Assessment Model of Pain” that includes qualitative and quantitative criteria, self-report, and third-person measures are important steps in development ^13^. A distinction must be made between differential perception, expression, and citation. Although ultimately only the patient is in pain, the patient’s own experience of pain and that of the paramedic will influence the assessment.

Assessing pain can be challenging, especially in emergencies and prehospital emergency medicine. On the other hand, the success of analgesia is in standardized data sets for quality assurance in emergency medicine one-dimensional and often reduced to the Numeric Rating Scale, such as with the need for prehospital analgesia from NRS ≥ 5, the success of analgesia is measured at patient handover and defined there at NRS < 5 or a reduction of ≥ 2 points^14^. This initially shows the discrepancy—between pain perception, pain processing, pain expression, and the correct derivation of suitable analgesic measures.

Due to the complexity and interdependencies involved, it is not surprising that healthcare professionals and patients may arrive at different assessments of patient pain^7^. This is especially true in emergencies, where relatively little is known about the frequency and prevalence of pain^15^. However, it is unclear whether paramedics and patients agree or differ in their assessment of pain in prehospital emergencies. This lack of knowledge highlights the need for further research in this area.

### Objectives

The study aimed to assess the disparities in pain ratings between patients and paramedics and identify the overall pain ratings and the factors influencing them in prehospital emergencies.

## Methods

### Study design

This prospective cross-sectional observational study utilized data from the rescue service quality assurance of the German Red Cross EMS in Reutlingen, Germany. The manuscript follows the RECORD-Statement (Reporting of Studies Conducted using Observational Routinely-collected Health Data) guidelines^16^.

### Setting

The rescue service covers an area of 1092.46 km^2^ with a population of 288,158. For several years, the EMS has implemented a competence system whereby paramedics are trained, qualified, and authorized to independently perform procedures, including analgesia, under physician supervision^17–19^. This advanced delegation is supported by comprehensive training, standardized operating procedures (SOPs), and regular competence checks, which are closely monitored by quality management ^20^.

### Participants

All patients who received treatment and were transported by paramedics, with some cases involving the participation of an emergency physician, were included in the study. Records with missing patient data were excluded from the analysis.

### Outcomes

The study’s primary endpoint was to examine the disparity in pain ratings between patients and paramedics. The secondary endpoints included: (a) patients’ subjective assessment of whether pain could be tolerated until hospital admission or if immediate pain control was necessary, (b) pain prevalence, and (c) factors influencing pain assessment by both patients and paramedics.

### Variables

The analysis in this study incorporated several variables, including patient age, sex, and pain parameters assessed using the Numeric Rating Scale (NRS). In addition to the NRS, it was possible to select in the data set whether the patient felt their pain was bearable or not, provided they were able to answer the question. Furthermore, the Glasgow Coma Scale (GCS), systolic blood pressure, heart rate, respiratory rate, and oxygen saturation (SpO_2_) were considered at the initiation of prehospital care. The city and type of municipality where the patient resided were also included as variables. Furthermore, the sex and age of the paramedics were documented for analysis purposes.

### Data sources

All data were collected during the prehospital phase as part of the electronic case documentation utilizing the NaProt/DocYou software developed by Pulsation IT (Berlin, Germany).

### Ethics

This study was conducted in accordance with applicable laws and guidelines. Regarding the consent of the data, please note the following: The study is based on the legally required routine data of the ambulance service, to which a question about pain was prospectively defined. The dataset is defined at the state level. The current EU GDPR defines that the lawfulness of processing is met when the “data subject has given consent”, or as in this case, “compliance with a legal obligation” (Art. 6 GDPR paragraph 1c) is given, as well as the “performance of a contract”, in this case, the treatment contract with the ambulance service (Art. 6 GDPR paragraph 1b). The legal obligation comes from the state rescue service law, insofar as no extra informed consent is necessary according to European/German law. Compliance with the legal requirements of the European Data Protection Regulation has been reviewed and approved by the Data Protection Officer. This research project has been reviewed and approved by the Ethics Committee of the Medical Faculty of the Eberhard Karls University and the University Hospital of Tübingen (approval number: 270/2022BO2).

### Statistical methods

Descriptive statistics were utilized to present metric scale variables, reported as mean ± standard deviation. Frequencies were expressed as absolute and relative numbers. Statistical significance was determined using two-tailed *P*-values with a threshold of < 0.05. The χ^2^-test was employed to examine categorical variables, while the t-test or single-factor analysis of variance (ANOVA) was used for independent samples with normally distributed data to assess differences. For the univariate analysis, logistic regression was used to identify factors influencing the difference in pain ratings between patients and paramedics. Statistical analyses were conducted using SPSS Statistics 29 (IBM, Armonk, NY, USA).

## Results

**Figure 1:**
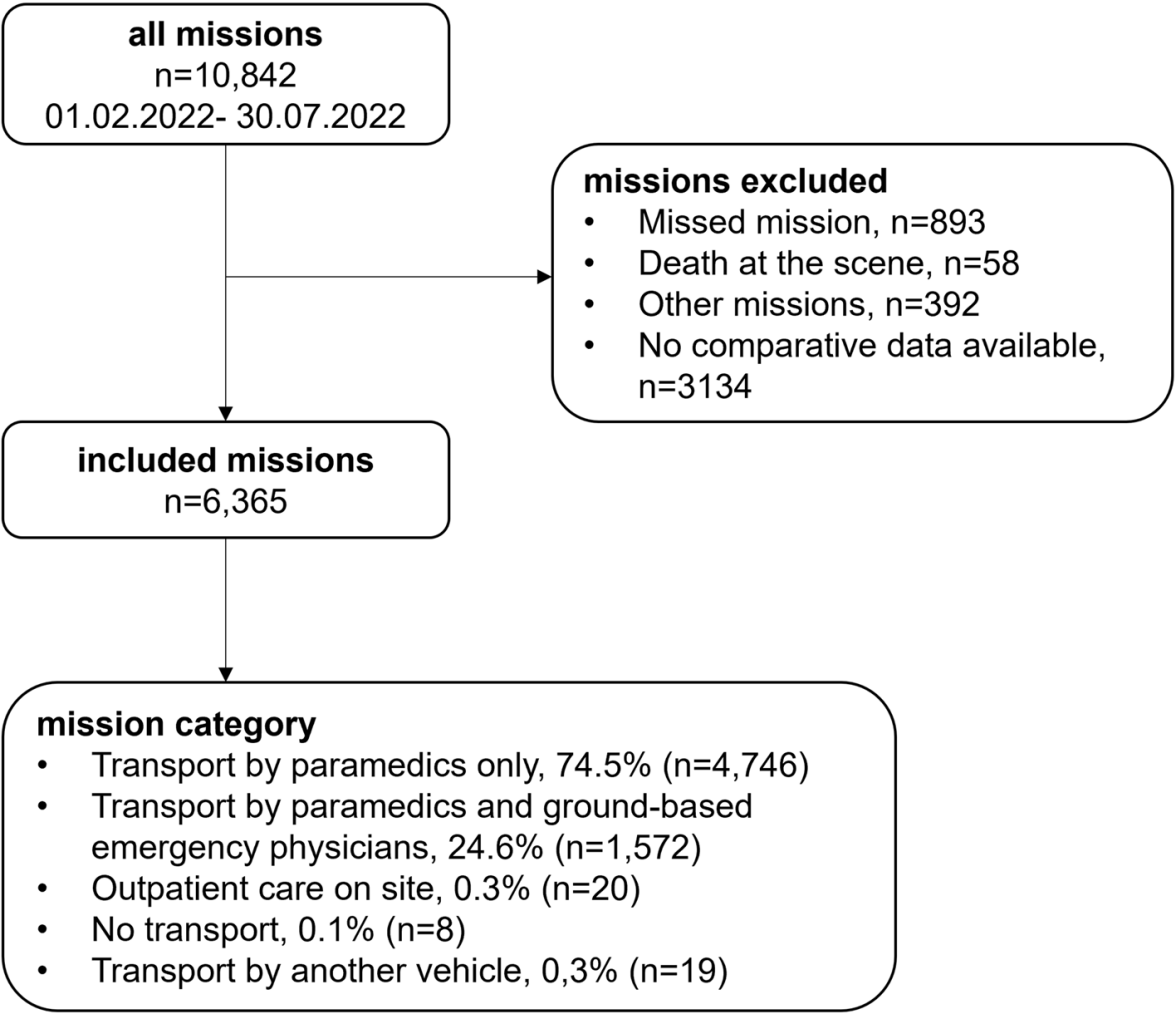
Patient inclusion criteria.

Between 1 February 2022, and 30 July 2022, a total of 6,365 patients were included in the study (Table 1). Most of these patients were transported by paramedics only (74.6%), followed by a combination of paramedics and ground-based emergency physicians (24.7%). A small percentage received further outpatient care on site (0.3%), while a negligible number of patients were deemed unfit for transport (0.1%) or required transfer to another rescue vehicle (0.3%) (Fig. 1**)**.

**Table 1:** Type of prehospital care and main characteristics of patients regarding differences between emergency physician-attended and paramedic-attended. Not included were 0.7% (n = 47) “other” missions.

Type of prehospital care	Transport with an emergency physician (ground-based)	Transport paramedic guided	*p*-Value
Count	1,572	4746	
Percentage	24.7%	74.6%	
Age, years, mean ± SD	58 ± 25	61 ± 25	< 0.001
Sex, female, n (%)	780 (49.6%)	2466 (52.0%)	0.273
Initial Patient pain, NRS, Mean ± SD	3 ± 4	2 ± 2	< 0.001
Unbearable pain, n (%)	440 (33.4%)	230 (5.2%)	< 0.001
Paramedic pain assessment NRS, Mean ± SD	3 ± 3	1 ± 2	< 0.001

The included patients had a mean age of 60 ± 26 years, with females accounting for 51.4% of the sample.

The attending paramedics had an average age of 32 ± 12 years (ranging from 20 to 65), with females comprising 26.8% of the group. The patients’ pain rating was reported as NRS 2.1 ± 2.8, whereas the paramedics’ pain rating was NRS 1.8 ± 2.5 (*p* < 0.001).

**Figure 2:**
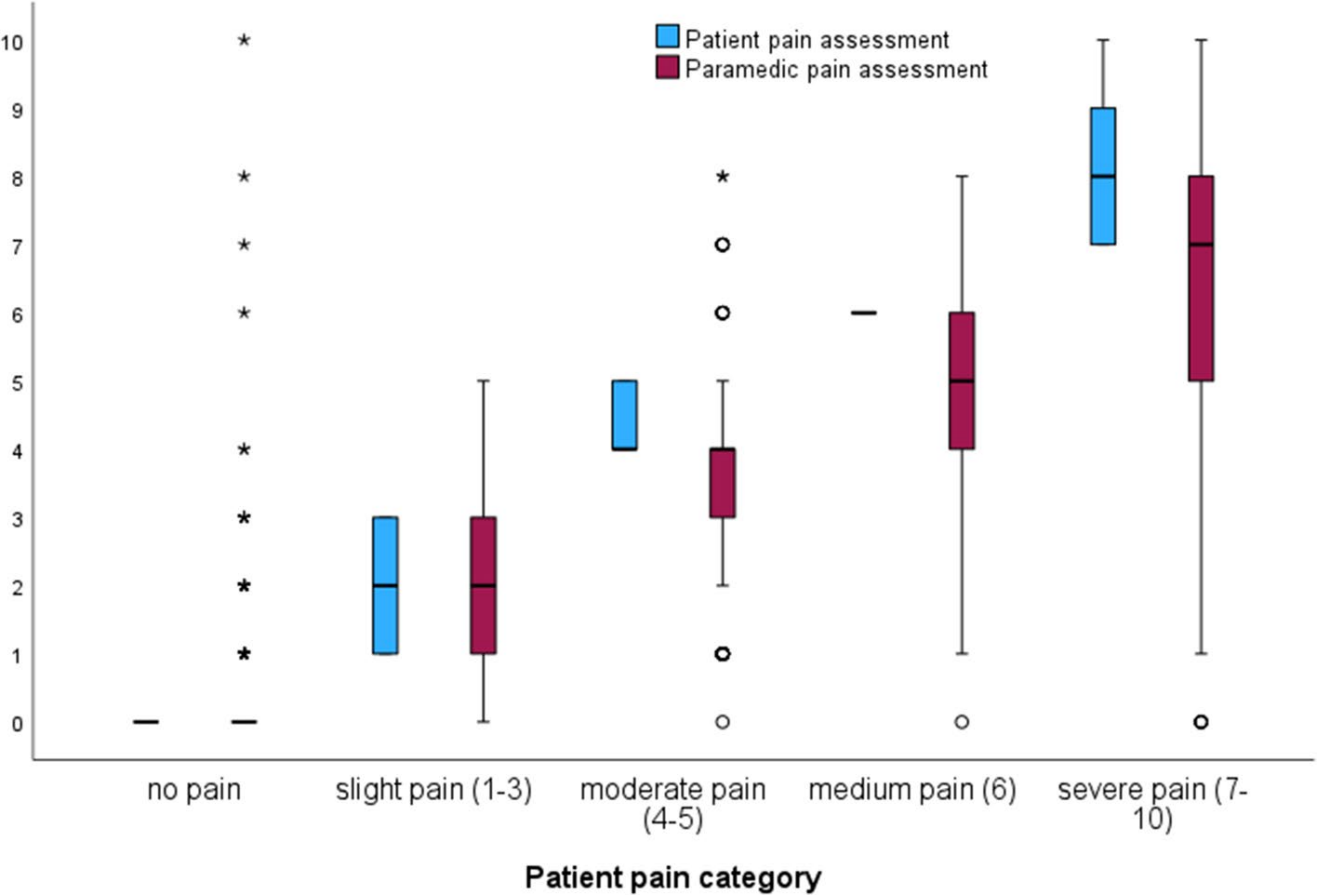
Comparison of patient and paramedic pain ratings based on pain categories. The figure shows box plots with median and interquartile ranges with whiskers. The difference in pain rating increased with pain severity. Slight pain category, the mean difference was 0.14 ± 0.51, moderate pain category, the mean difference was 0.73 ± 1.12; medium pain category, the mean difference was 1.29 ± 1.54; severe pain category, the mean difference was 1.71 ± 2.22, *p* < 0.001.

The patients’ pain ratings had a mean of NRS 2.1 ± 2.8 (on a scale of 0–10), with a median of 0. Categorizing the pain levels, the distribution was as follows: 50.3% (n = 2851) reported no pain, 25.1% (n = 1422) had mild pain (NRS 1–3), 9.9% (n = 562) experienced moderate pain (NRS 4–5), 3.0% (n = 168) had medium pain (NRS 6), and 11.8% (n = 669) suffered from severe pain (NRS 7–10). The mean pain rating given by the paramedics was 1.8 ± 2.4 (on a scale of 0–10), with a median of 1, compared to the patient’s pain rating (*p* < 0.001). When specifically asked, 11.8% (n = 678) of patients reported unbearable pain. Notably, as pain category and intensity increased, there were significant divergences between the pain ratings of patients and paramedics (Fig. 2).

When considering only patients who self-reported a NRS score greater than zero, the patient pain rating was recorded as NRS 4.2 ± 2.7, while the paramedic pain rating was NRS 3.6 ± 2.4 (*p* < 0.001). Subjectively unbearable pain was reported by 11.8% (n = 751) of patients. In cases of unbearable pain, patients reported an NRS score of 7.7 ± 1.8 (range 0–10, median 8), compared to bearable pain with an NRS score of 3.3 ± 2.0 (range 0–10, median 0) (*p* < 0.001).

Comparing the pain scores of patients based on their place of residence or the size of the area, there were no significant differences in mean pain scores (*p* = 0.691).

**Figure 3:**
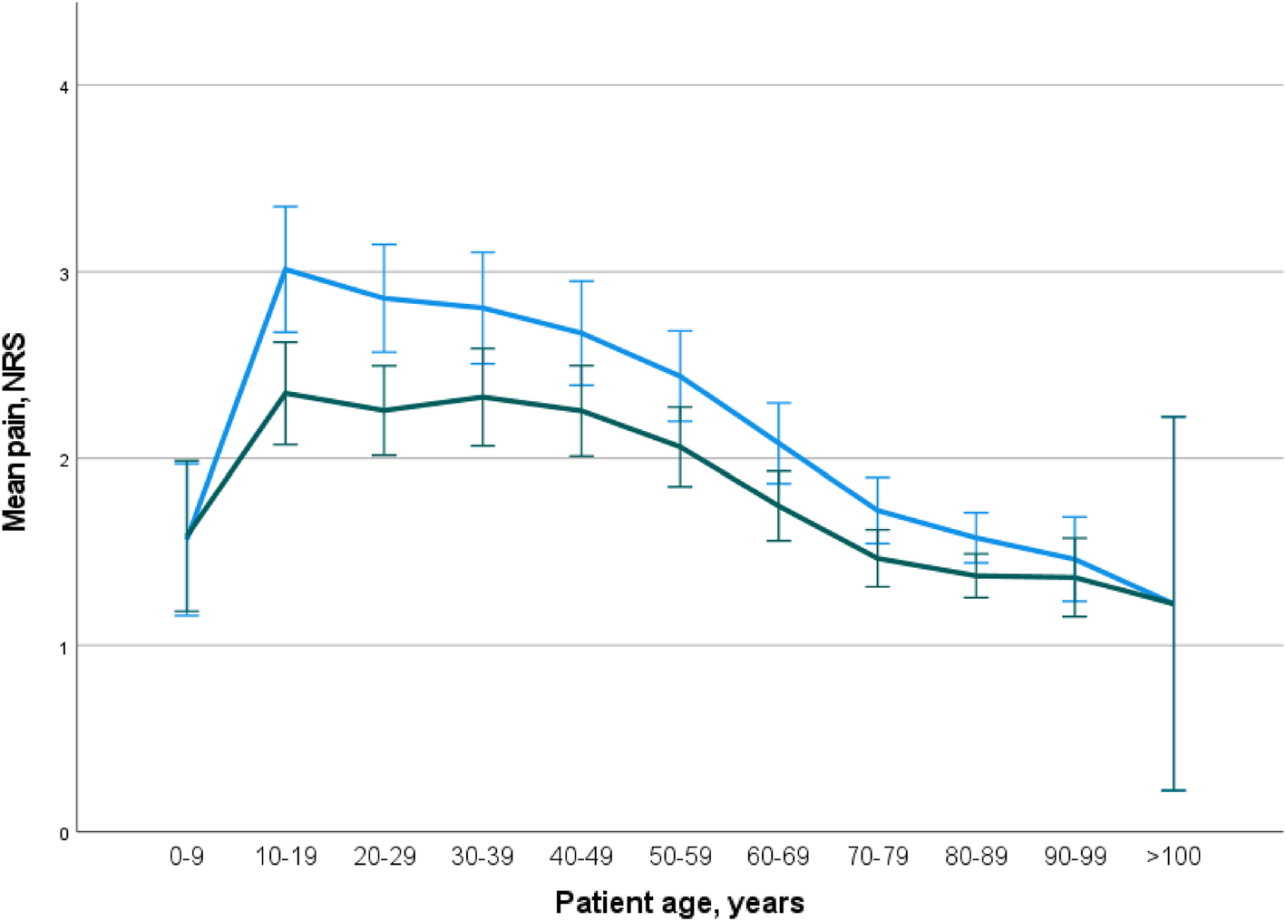
Pain assessment of patients (blue) and paramedics (green) in relation to age. Data with mean and 95% confidence interval. Age groups on the x-axis and NRS on the y-axis.

The analysis of pain assessment in relation to patient age reveals a notable difference between patients and professionals, particularly within the younger age group of 10 to 30 years (*p* < 0.001). Subsequently, the pain ratings provided by patients and professionals gradually converge as age increases (Fig. 3).

**Figure 4:**
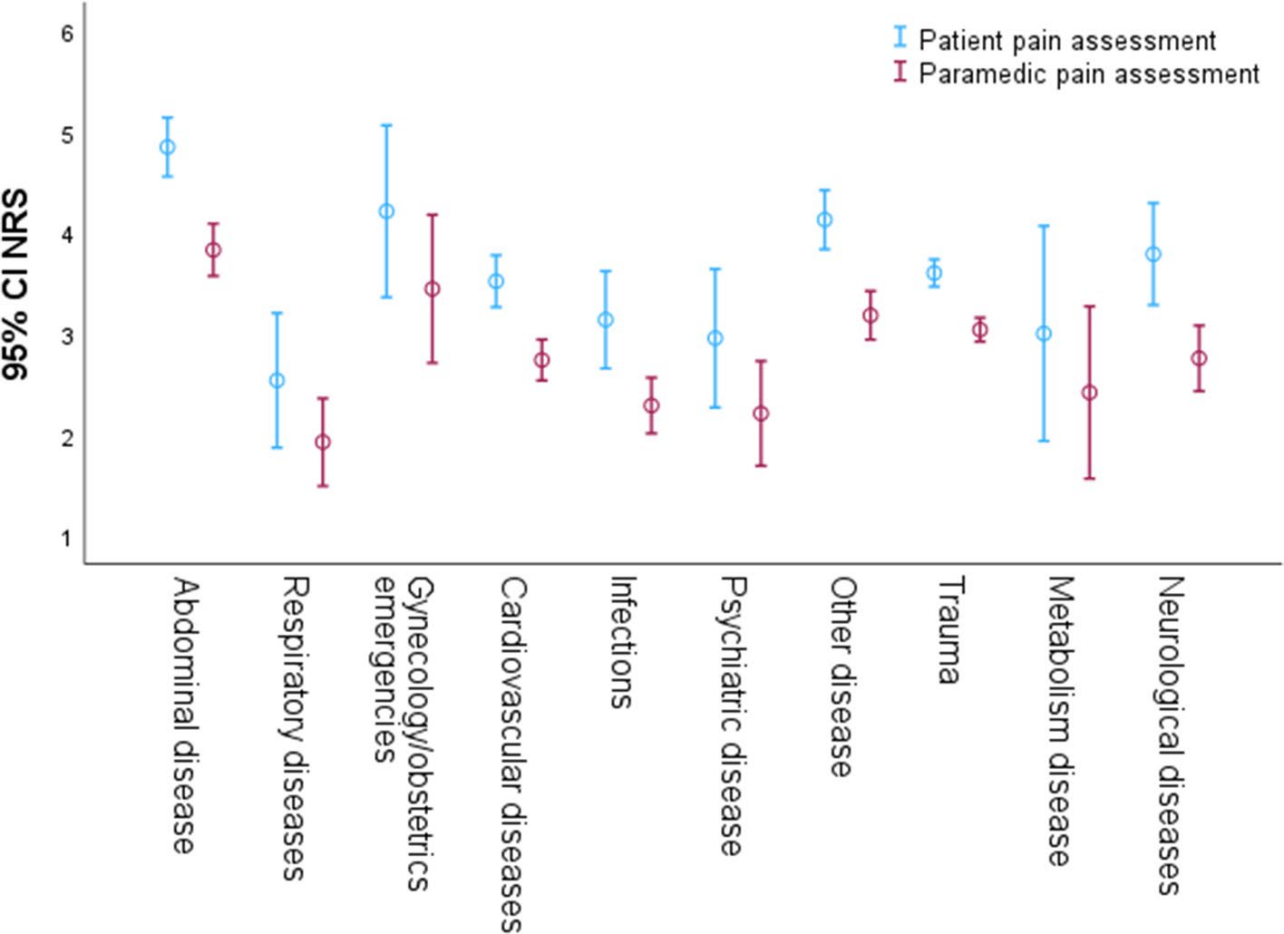
Pain assessment of paramedics and patients, listed by diagnosis groups on the x-axis and pain on the y-axis (mean values with 95% confidence interval (CI)). Pain in the abdominal pain and traumatic pain groups differed significantly (*p* < 0.001).

When examining pain assessment by female paramedics, the pain scores (NRS > 0) for female patients were evaluated as 3.2 ± 2.2 (reported pain: 4.1 ± 2.7), while male patients received scores of 3.5 ± 2.5 (reported pain: 4.1 ± 2.7) (*p* = 0.119). The difference in reported pain (NRS > 0) between female and male patients was 4.1 ± 2.7 versus 4.1 ± 2.7, respectively (*p* = 0.601). Similarly, when male paramedics assessed pain (NRS > 0), female patients received scores of 3.6 ± 2.4, compared to male patients who received scores of 3.6 ± 2.4 (*p* = 0.610). The difference in reported pain (NRS > 0) between female and male patients was 4.3 ± 2.7 versus 4.3 ± 2.7, respectively (p = 0.879). Notably, significant differences are observed for abdominal diseases (*p* < 0.001) and trauma (*p* < 0.001), highlighting pronounced disparities in pain presentation among these conditions (Fig. 4).

However, the regression analysis indicates that patient and paramedic age are the only variables that significantly affect the disparity between patient and paramedic pain ratings (Table 2).

**Table 2:** Regression analysis with the independent variable pain difference.

	Non-standardized regression coefficient	Standard error	*p*-Value	95.0% Confidence interval
Patient age	− 0.008	0.001	** < 0.001**	− 0.011–0.005
Patient sex	− 0.058	0.059	0.327	− 0.173–0.058
Paramedic sex	− 0.05	0.07	0.479	− 0.187–0.088
Paramedic age	− 0.005	0.003	**0.042**	− 0.010–0.00
Community Category	0.001	0.016	0.927	− 0.030–0.033
Glasgow Coma Scale	0.079	0.088	0.369	− 0.093–0.251
Systolic Blood pressure	0	0.001	0.808	− 0.002–0.002
Oxygen saturation	0.008	0.005	0.119	− 0.002–0.018
Heart rate	− 0.002	0.002	0.253	− 0.005–0.001
Respiratory rate	− 0.002	0.006	0.697	− 0.014–0.010
Diagnosis category	− 0.018	0.011	0.095	− 0.039–0.003

Significant values are in [bold].

## Discussion

This study addresses the analysis of prehospital pain, including perspectives of both patients and paramedics, as well as the implications for therapy and quality assurance. It is worth noting that the difference in pain ratings between patients and paramedics is not as significant as previously suggested.

### Rating of pain by patients and paramedics

The prevalence of pain in emergency services highlights the significance of this matter. However, the subjective nature of pain poses a challenge. Numerous publications have examined pain and pain ratings, primarily from the perspective of hospital staff, and many of them indicate that healthcare workers tend to underestimate patients’ pain^21–25^. Nevertheless, the ultimate question remains: are healthcare professionals underestimating patients’ pain, or are patients potentially overexpressing their pain?

**Figure 5:**
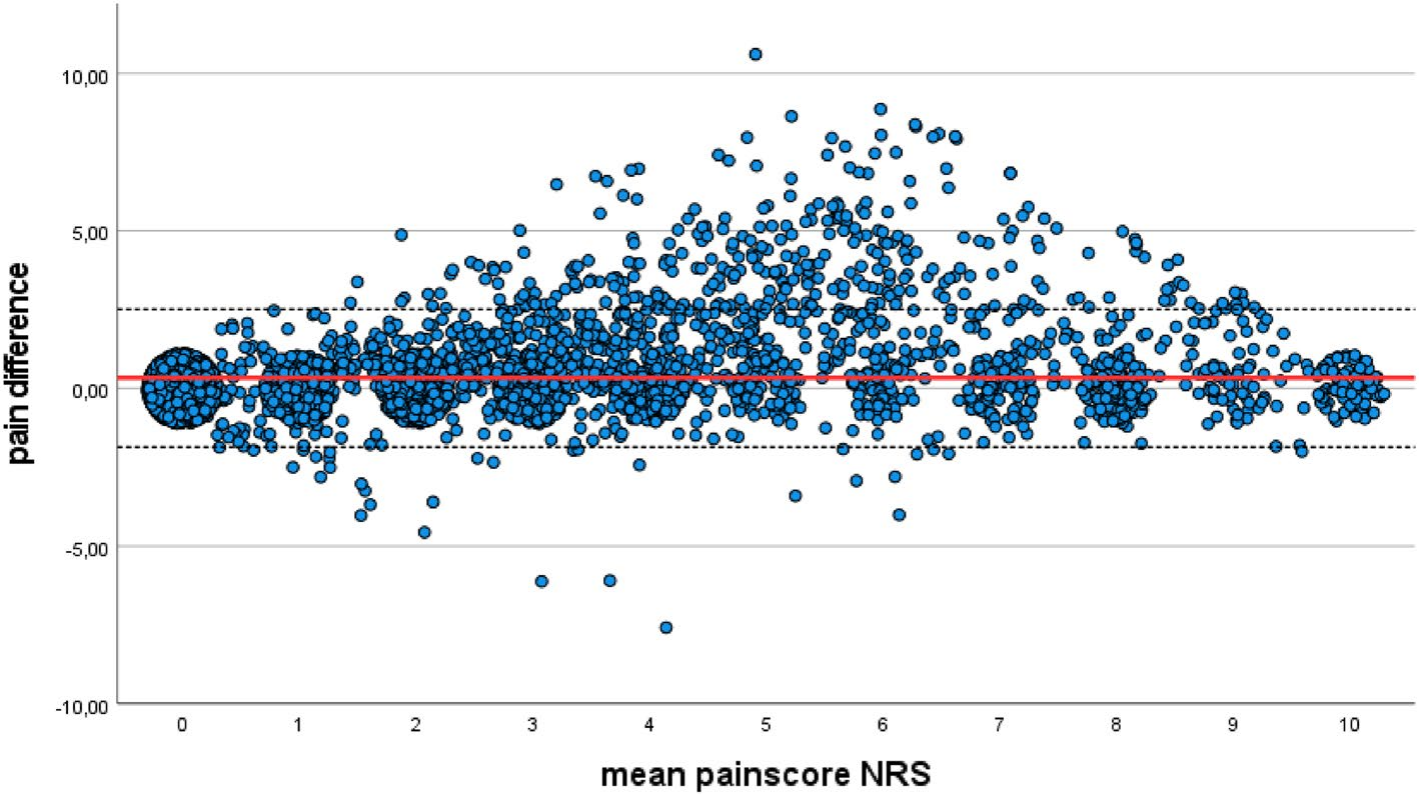
Jittered Altman-Plant diagram showing the plot of pain difference (y-axis) and pain scale NRS (x-axis). In red, the mean pain difference and dashed the 95% confidence interval.

The regression analysis shows a significant impact of both patient and paramedic age on the divergent pain ratings. Figure 5 demonstrates that particularly younger patients and presumably younger paramedics exhibit varying perceptions of pain. The concept of pain experience is particularly relevant in this context. Younger patients may have experienced less pain compared to older patients, including acute pain from accidents, surgery, and chronic pain conditions. Therefore, their maximum pain rating may be based on their limited experiential horizon. In a systematic review of pain management in the emergency department, Sampson et al. emphasized that pain management is rooted in experience rather than mere knowledge^24^. This finding aligns with our observations regarding the influence of age.

### Prevalence of prehospital pain

The dataset shows that 49.7% of the patients had at least mild pain. Furthermore, patients who were transported by an emergency physician initially reported higher pain levels (NRS ground physician: 3 ± 4) than those accompanied solely by a paramedic (NRS: 1 ± 2). Within the entire dataset, 11.8% reported unbearable pain. The literature presents varying prevalence rates for prehospital pain, ranging from 20 to 70%^26^. In a study by Galinski et al., acute pain was reported in 42% of over 2000 patients^27^. These findings are consistent with our results. It is important to note that the interpretation of ‘severe pain’ as NRS > 6, is subjective and contextual. Furthermore, the data indicate that patients who received care and transportation from paramedics and emergency physicians experienced more severe pain than those transported by paramedics alone. This suggests that patients attended by emergency physicians may have more severe injuries or illnesses, although the specific circumstances of each case undoubtedly influence this interpretation.

Our data shows no statistically significant difference in pain ratings between female and male patients. Additionally, regression analysis indicates that the gender of the patient or paramedic does not significantly affect the disparity in patient and paramedic ratings. This finding is significant, because it challenges the commonly held belief that women are more sensitive to pain and more susceptible to clinical pain due to biological, psychosocial factors, and gender stereotypes^28^.

Nevertheless, it is important to acknowledge that the underlying cause of pain contributes to the intensity of pain experienced and the variations in pain assessment.

When considering the causes of pain, it becomes necessary to consider that visibly apparent sources of pain, such as dislocated fractures or similar injuries, can influence the paramedic’s perception due to a halo effect. Conversely, non-verbal expressions of pain may also have comparable effects. In our study, a notable difference in pain assessment among patients with abdominal pain is understandable but less comprehensible in patients with injuries. The variability of injuries could be one possible explanation for this disparity. In the dataset used, injuries are presented in an aggregated manner, which may result in a high proportion of minor injuries. This could limit the discriminatory power of the analysis.

It is important to recognize that the experience of pain for patients and the assessment of pain by physicians or paramedics can be influenced by their respective cultural backgrounds. Studies investigating the perception and experience of pain across different cultures have demonstrated variations, particularly in the emotional aspect of pain and the extent to which individually learned and culturally influenced coping mechanisms are employed^29^. Therefore, in assessing pain by paramedics and physicians, one’s sociocultural perspective, including expectations regarding pain experiences and expressions, often comes into play, as intercultural aspects are typically not incorporated in training or studies^29^.

While it is undoubtedly crucial to prioritize the patient’s needs, it is also essential to carefully consider the efficacy, potential adverse effects, and associated risks of pharmacological analgesia. In this context, it is important to consider the principle of proportionality. Professionals should focus on pain management and make decisions about analgesia based on the patient’s pain indication and overall clinical impression, rather than inquiring about patients’ desire for analgesics, which they may not fully understand^31^.

Efforts to objectify pain continue to evolve. In an attempt to predict pain levels, Bendall et al. explored using vital signs^33^. They found a weak correlation between respiratory rate and initial pain. However, there was expected to be a significant increase in pain when the respiratory rate exceeded 25/min, or the heart rate exceeded 100/min. Nevertheless, our study was unable to replicate these findings. This discrepancy could be attributed to the fact that, in our experience, the respiratory rate estimation is often imprecise. Additionally, in our study, blood pressure and heart rate were not predictive of severe pain.

### Limitations

This study’s common limitation in routine data analysis is that not all relevant factors are encompassed in the present dataset. For instance, ethnic or sociocultural characteristics and indications of cognitive limitations in patients, such as dementia, were not included. Additionally, within the electronic documentation, there was the possibility to indicate “not assessable” for pain, meaning only assessable pain data were included. However, it is important to note that this approach is not entirely selective.

## Conclusion

This study shows that patients consistently report higher pain scores than paramedics would rate them. The biggest influence on this difference appears to be the age of the patient and the paramedic. Given that almost half of patients in the EMS suffer from pain, these results underline the importance of integrating pain assessment and appropriate analgesic strategies as a mandatory part of the EMS.

## Data Availability

Complete datasets are available from the date of article publication by the Corresponding author, to investigators who provide an IRB letter of approval.
